# Psychiatric disorders associated with increased risk of colorectal cancer in the UK biobank cohort

**DOI:** 10.1038/s41598-025-31083-1

**Published:** 2026-01-13

**Authors:** Zijing Wang, Tingxi Zhu, Litao Huang, Xian Zhang, Xiuhe Lv, Xiaoshuang Zhang, Li Yang, David Kerr, Jinlin Yang

**Affiliations:** 1 Department of Gastroenterology and Hepatology&, West China Hospital, Sichuan University-University of Oxford Huaxi Joint Centre for Gastrointestinal Cancer, Sichuan University, Chengdu, 610000 China; 2 Department of Gastroenterology and Hepatology and Biomedical Big Data Center, West China Hospital, Sichuan University, Chengdu, 610000 China; 3 Nuffield Division of Clinical Laboratory Sciences, Radcliffe Department of Medicine, University of Oxford, John Radcliffe Hospital, Oxford, OX3 9DU UK; 4 Department of Clinical Research Management, West China Hospital, Sichuan University, Chengdu, 610000 China; 5 Med-X Center for Informatics, Sichuan University, Chengdu, 610000 China; 6 Department of Gastroenterology and Hepatology, West China Hospital, Sichuan University-University of Oxford Huaxi Joint Centre for Gastrointestinal Cancer, Sichuan University, Chengdu, 610000 China

**Keywords:** Psychiatric disorders, Colorectal cancer, Cohort study, UK biobank, Cancer, Diseases, Oncology, Psychology, Psychology, Risk factors

## Abstract

Psychiatric disorders are associated with several cancers. However, data on the association between different types of psychiatric disorders and colorectal cancer (CRC) are scarce. The aim of this study was to determine whether there is an association between psychiatric disorders and CRC using UK Biobank. We excluded participants with a previous diagnosis of cancer (except non-melanoma skin cancer) prior to baseline and conducted a matched cohort study with new-onset psychiatric disorder patients after recruitment as the exposure group and matched non-psychiatric disorder individuals as the reference group. Through UK Biobank, we obtained clinical diagnoses for the psychiatric-disorder patients from their hospital records. The Cox proportional hazards model was used to estimate the hazard ratios (HRs) of CRC risk after diagnosis of a psychiatric disorder. We identified 29,769 individuals with psychiatric disorders. During follow-up (median duration of 5.69 [IQR: 5.43] years), 190 cases of CRC were identified in the psychiatric patients (1.13 per 1000 person-years), compared with 921 cases in the reference individuals (0.53 per 1000 person-years). Individuals with clinically confirmed psychiatric disorders were associated with an elevated hazard of CRC (HR, 1.93 [95% CI, 1.64–2.27]). The risk of CRC in psychiatric patients is higher in those with multiple psychiatric disorders than single (HR 2.53 [95% CI, 1.90–3.37] vs. 1.78 [95%CI, 1.48–2.13], *p* < 0.05). Our findings suggest: patients with psychiatric disorders were associated with an elevated hazard of CRC. These findings highlight the need for measures to decrease the risk of CRC in individuals with psychiatric disorders, such as monitoring and early intervention.

**Supplementary Information:**

The online version contains supplementary material available at 10.1038/s41598-025-31083-1.

## Introduction

Colorectal cancer (CRC) is the third most common cancer worldwide. More than 1.8 million new cases are reported each year, ranking third in incidence and second in mortality among all types of cancers^1,2^. In recent decades, its incidence and mortality rate have steadily increased in China, leading to markedly elevated healthcare use for individuals and medical costs for society^3^. Accordingly, identifying risk factors associated with CRC is a necessary step towards decreasing its incidence and associated healthcare burden.

Psychiatric disorders, particularly depression and anxiety, are common public health problems that are associated with a range of chronic health conditions, including coronary heart disease, diabetes, and cancer^4^. According to a report by the World Health Organization, almost one in seven people worldwide live with a mental health condition^5^. Furthermore, the prevalence of psychiatric disorders differs among women and men^6,7^. Potential causative links between psychiatric disorders and cancer are psychological stress and poor health behaviours, which are known to cause neuroendocrine, immunological, and behavioural changes that increase the risk of cancer and promote its development^8^. In addition, psychological disorders can impact screening compliance and thus delay or prevent intervention. Although links between CRC and certain psychiatric disorders (e.g., depression) are well established, most previous studies on psychiatric disorders (i.e. depression or anxiety) combined with CRC incidence were limited by moderate sample size or gender-limited samples (i.e. research female or male only) and the results are also inconsistent, reporting positive or null findings^9,10^. Thus, the association between CRC and psychiatric disorders remains controversial and unclear. Furthermore, to our best knowledge, there has been no comprehensive assessment of the link between different types of clinically confirmed pre-existing psychiatric disorders and CRC susceptibility and fatality using longitudinal data. Therefore, more detailed and accurate information is required to better inform both clinical and psychological interventions.

UK Biobank is a largescale prospective cohort study including over 500,000 participants. It is a rich source of information on phenotypes relevant to psychiatric disorders, sociodemographic, socioeconomics, and lifestyle factors, and it contains a large number of recorded CRC cases. Accordingly, we used it to investigate whether psychiatric disorders increased the risk of CRC incidence and mortality.

## Materials and methods

### Study population

The UK Biobank is a prospective cohort study of 502,415 participants aged 37–73 years from England, Scotland, and Wales recruited between 2006 and 2010. At the baseline recruitment visit, participants were asked to complete a self-administered questionnaire that included questions on sociodemographic characteristics (including age, sex, race, educational status, and Townsend deprivation index), health and medical history (including cancer screening), lifestyle exposures (including smoking habits, dietary intake, and alcohol consumption), family history, physical measurements, medication use, and other health-related factors. At recruitment, all the UK Biobank participants gave written informed consent for participation and follow up.

We excluded participants with a previous diagnosis of cancer (except non-melanoma skin cancer) prior to baseline, and excluded participants who had undergone (or were not sure whether they had undergone) bowel cancer screening prior to recruitment, as the incidence of CRC is dramatically decreased by screening efforts to detect and remove premalignant lesions^11^. Additionally, participants who had withdrawn from UK Biobank were excluded. We created an exposed cohort of all individuals who have received their first diagnosis of a psychiatric disorder between July 2006 and December 2021 (updated to December 15, 2021) and excluded patients with a history of cancer diagnosis (except nonmelanoma skin cancer) prior to their psychiatric disorder. Furthermore, we matched each patient with psychiatric disorders by gender and birth year to 10 comparators from the UK Biobank, which means comparators would have the same gender and birth year as the original patient. Matched reference individuals must be alive and have no history of psychiatric disorders and cancer (except non-melanoma skin cancer) at the diagnosis date of the psychiatric disorder of the index patient^12^.

## Psychiatric disorders

We retrieved information about the psychiatric disorder diagnoses from UK Biobank inpatient hospital data. Psychiatric disorders were defined as those leading to a hospital admission and a diagnosis of a psychiatric disorder according to the International Classification of Diseases (ICD) 10th Edition (ICD-10) codes F10–F99 and ICD 9th Edition (ICD-9) codes 295–319. We further classified psychiatric disorders into depression (ICD-10: F32–F33; ICD-9: 296.1, 300.4, and 311), anxiety (ICD-10 F40–F41; ICD-9 300.0 and 300.2), stress-related disorder (ICD-10 F43; ICD-9 308 and 309), substance abuse (ICD-10 F10–F19; ICD-9 291 and 303–305), psychotic disorders (ICD-10 F20–F29; ICD-9 295, 297, and 298), and other psychiatric disorders.

## Ascertainment of cancer outcome and cancer-specific mortality

Cases of cancer within the UK Biobank cohort had been identified through a link with the national cancer registries (UKB data category 100092). CRC was defined by ICD codes (ICD-10:C18-20; ICD-9:153–154). Colon cancer was defined as C18 (ICD-10) and 153 (ICD-9) and rectal cancer as C19 and C20 (ICD-10) and 154 (ICD-9). Cancer-specific mortality was ascertained through primary cause of death by linkage to national death registries (UKB data category 40001).

## Covariates

Data on sociodemographic characteristics (birth year, sex, and ethnicity), socioeconomic factors (Townsend deprivation index, educational attainment, and annual household income), and lifestyle factors (smoking, drinking status, and meat consumption) were collected by questionnaire at baseline. Townsend deprivation index is a measure of the material deprivation within a population which was first described by the sociologist Peter Townsend in 1988^13^. Body mass index (BMI) was calculated from height and weight measurements taken during the subject’s initial visit to an assessment centre. Factors that could affect CRC susceptibility or course, such as family history, diabetes mellitus, hormone replacement therapy (female only), and aspirin intake, were considered as potential confounders, in line with the literature^14^. All covariates were divided into three groups to meet subsequent analysis requirement: sociodemographic group including birth year, sex, ethnicity, Townsend deprivation index, education attainment and household income; health status group including BMI, family history, aspirin intake, diabetes mellitus and HRT; behavioural group including smoking, drinking status and meat consumption. A few individuals (0.13%) had missing values in Townsend deprivation index, and these were imputed by the median among other patients.

### Statistical analysis

The incidence rate of CRC and cancer-specific mortality were calculated with 95% confidence intervals (CIs) and expressed as number of events per 1000 person-years. Cox proportional hazard models were used to estimate the hazard ratios (HRs) for psychiatric disorder on CRC risk with 95% CI.

For analysis of CRC incidence (based on the cancer register), the follow-up period started from the index date (date of psychiatric disorder diagnosis in the exposed cohort; the unexposed patient took the same index date as their matched case) to the date of the first diagnosis of CRC, any other primary cancer (except non-melanoma skin cancer), death, lost-to-follow-up, or the end of the study (updated to December 15, 2021), whichever occurred first. For CRC-mortality analysis (based on the cause of death register), CRC incidence was disregarded, follow-up until death, lost-to-follow-up, a new diagnosis of a psychiatric disorder, or the end of the study (updated to December 15, 2021), whichever occurred first. Follow-up of reference individuals also ended if they developed a psychiatric disorder. The follow-up of unexposed individuals was stopped contributing reference person-­time if they were later diagnosed as having psychiatric disorders. Follow-up dates between index dates and any endpoint were treated as survival time, and selected covariates were treated as potential risk factors.

The main Cox model was progressively built on the entire study population. It started as a crude model with psychiatric disorders only and then gradually added sociodemographic group covariates, health status group covariates, and behavioural group covariates. The full model was adjusted by birth year, gender, ethnicity (Black, White, Chinese, etc.), Townsend deprivation index, education attainment (College or University degree, A levels/AS levels or equivalent, etc.), annual household income (< 18,000, 18,000–30,999, 31,000–51,999, etc.), BMI, smoking and drinking status (never, ever, unknown), meat consumption (less than twice a week, 2–4 times a week, more than 4 times a week, unknown), family history of CRC (yes or no), aspirin intake (yes or no), diabetes mellitus (yes, no, unknown), and hormone replacement therapy treatment (yes, no, unknown; female only). We added the sex and cancer location as interaction terms in the full model to statistically explore the interaction effect on the relationship between psychiatric disorders and CRC.

For subgroup analysis, patients were divided into several groups based on the basis of their gender (male or female), type of psychiatric disorder (depression, anxiety, stress-related disorder, substance abuse, or psychotic disorder), and type of CRC (colon cancer or rectal cancer). HRs for the association between psychiatric disorder and CRC were calculated within all groups. Subsequential Cox models were adjusted by the same covariates as those used for the full model (hormone replacement therapy was only considered for females). Furthermore, we explored the associations between CRC incidence and the number of psychiatric disorders (1 or ≥ 2).

A two-tailed p-value less than 0.05 was considered statistically significant. All analyses were performed using R (version 3.6.1) software. All methods were carried out in accordance with the relevant guidelines and regulations.

## Results

### Baseline characteristics

**Fig. 1:**
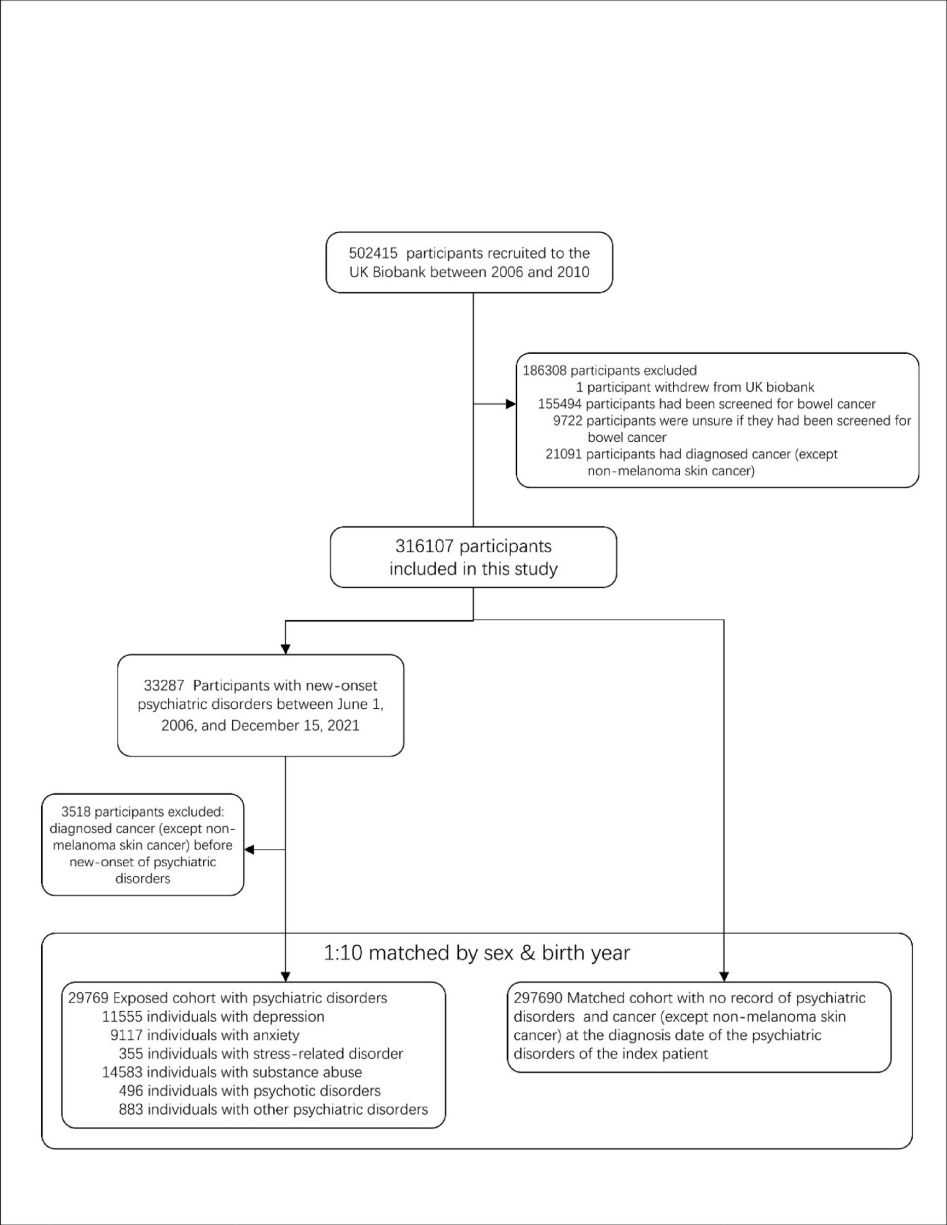
Flow Chart of Study design.

Of the 502,415 UK Biobank participants, 186,308 people were excluded from the study in accordance with the exclusion criteria, leaving 316,107 eligible participants for further analyses (Fig. 1). Among them, 29,769 patients that had their first psychiatric disorder diagnosed between June 1, 2006 and December 15, 2021were identified. Then, 297,690 matched unexposed individuals who had no history of psychiatric disorders and cancer (except non-melanoma skin cancer) at the diagnosis date of the index patient were included as the reference group, which is essential for minimizing confounding effects arising from pre-existing malignancies and their treatments.

Of all 29,769 patients, 46.18% were male, and their median age at first psychiatric diagnosis was 62 (IQR: 14) years. Compared with patients without psychiatric disorders, we found higher proportions of patients with psychiatric disorders who were smokers, had higher BMIs, consumed more red meat and aspirin, received hormone replacement therapy treatment, had a family history of CRC, and had lower socioeconomic and educational statuses (Table 1). We also found higher proportions of patients with psychiatric disorders who had a history of severe somatic comorbidity, such as diabetes, compared with those without psychiatric disorders.

**Table 1:** Characteristics of the study cohorts.

Charcteristic	Persons with Psychiatric disorders	Persons without Psychiatric disorders
	29,769	297,690
**Age group**, **No. (%)**		
<60	12,290 (41.28)	122,900 (41.28)
≧60	17,479 (58.72)	174,790 (58.72)
**Gender, ** **No. (%)**		
male	13,747 (46.18)	137,470 (46.18)
female	16,022 (53.82)	160,220 (53.82)
**Race or Ethnicity**, **No. (%)**		
White	27,859 (93.58)	278,255 (93.47)
Black	539 (1.81)	5464 (1.84)
Asian	686 (2.30)	8172 (2.75)
Others	542 (1.82)	4822 (1.62)
Unknown	143 (0.48)	977 (0.33)
**townsend**, **median [25th**,** 75th percentile]**	−1.14 [−3.12, 2.14]	−2.20 [−3.68, 0.37]
**Educational Attainment**, **No. (%)**		
College or University degree	6917 (23.24)	100,851 (33.88)
A levels/AS levels or equivalent	3049 (10.24)	34,650 (11.64)
O levels/GCSEs or equivalent	6374 (21.41)	62,993 (21.16)
CSEs or equivalent	2323 (7.80)	18,217 (6.12)
NVQ or HND or HNC or equivalent	2331 (7.83)	18,756 (6.30)
Other professional qualifications	1388 (4.66)	14,172 (4.76)
None of the above	6594 (22.15)	42,751 (14.36)
Prefer not to answer	793 (2.66)	5300 (1.78)
**Household Annual Income**, **No. (%)**		
Less than 18,000	8274 (27.79)	48,022 (16.13)
18,000 to 30,999	6535 (21.95)	61,526 (20.67)
31,000 to 51,999	5895 (19.80)	71,767 (24.11)
52,000 to 100,000	3524 (11.84)	59,460 (19.97)
Greater than 100,000	638 (2.14)	15,204 (5.11)
Do not know	1719 (5.77)	11,053 (3.71)
Prefer not to answer	3184 (10.70)	30,658 (10.30)
**Body mass index (BMI)**, **No. (%)**		
<18.5	223 (0.75)	1350 (0.45)
18.5–24.9	8436 (28.34)	99,874 (33.55)
25.0–29.9.0.9	11,797 (39.63)	125,796 (42.26)
≧30.0	9030 (30.33)	69,324 (23.29)
Unknown	283 (0.95)	1346 (0.45)
**Smoking Status**, **No. (%)**		
Ever	19,226 (64.58)	122,577 (41.18)
Never	10,406 (34.96)	174,141 (58.50)
Unknown	137 (0.46)	972 (0.33)
**Drinking Status**, **No. (%)**		
Ever	28,384 (95.35)	284,186 (95.46)
Never	1324 (4.45)	13,250 (4.45)
Unknown	61 (0.20)	254 (0.09)
**Meat Consumption**, **No. (%)**		
Less than twice a week	16,720 (56.17)	180,459 (60.62)
2–4 times a week	11,264 (37.84)	104,681 (35.16)
More than 4 times a week	1765 (5.93)	12,423 (4.17)
Unknown	20 (0.07)	127 (0.04)
**Family History**, **No. (%)**		
Yes	2721 (9.14)	25,717 (8.64)
No	27,048 (90.86)	271,973 (91.36)
**Aspirin Intake**, **No. (%)**		
Yes	5133 (17.24)	38,135 (12.81)
No	24,636 (82.76)	259,555 (87.19)
**Hormone Replacement Therapy**, **No. (%)**		
Yes	6402 (21.51)	49,631 (16.67)
No	9547 (32.07)	110,068 (36.97)
Unknown	13,820 (46.42)	137,991 (46.35)
**Diabetes Mellitus**, **No. (%)**		
Yes	2180 (7.32)	12,879 (4.33)
No	27,476 (92.30)	284,139 (95.45)
Unknown	113 (0.38)	672 (0.23)

## CRC incidence

**Fig. 2:**
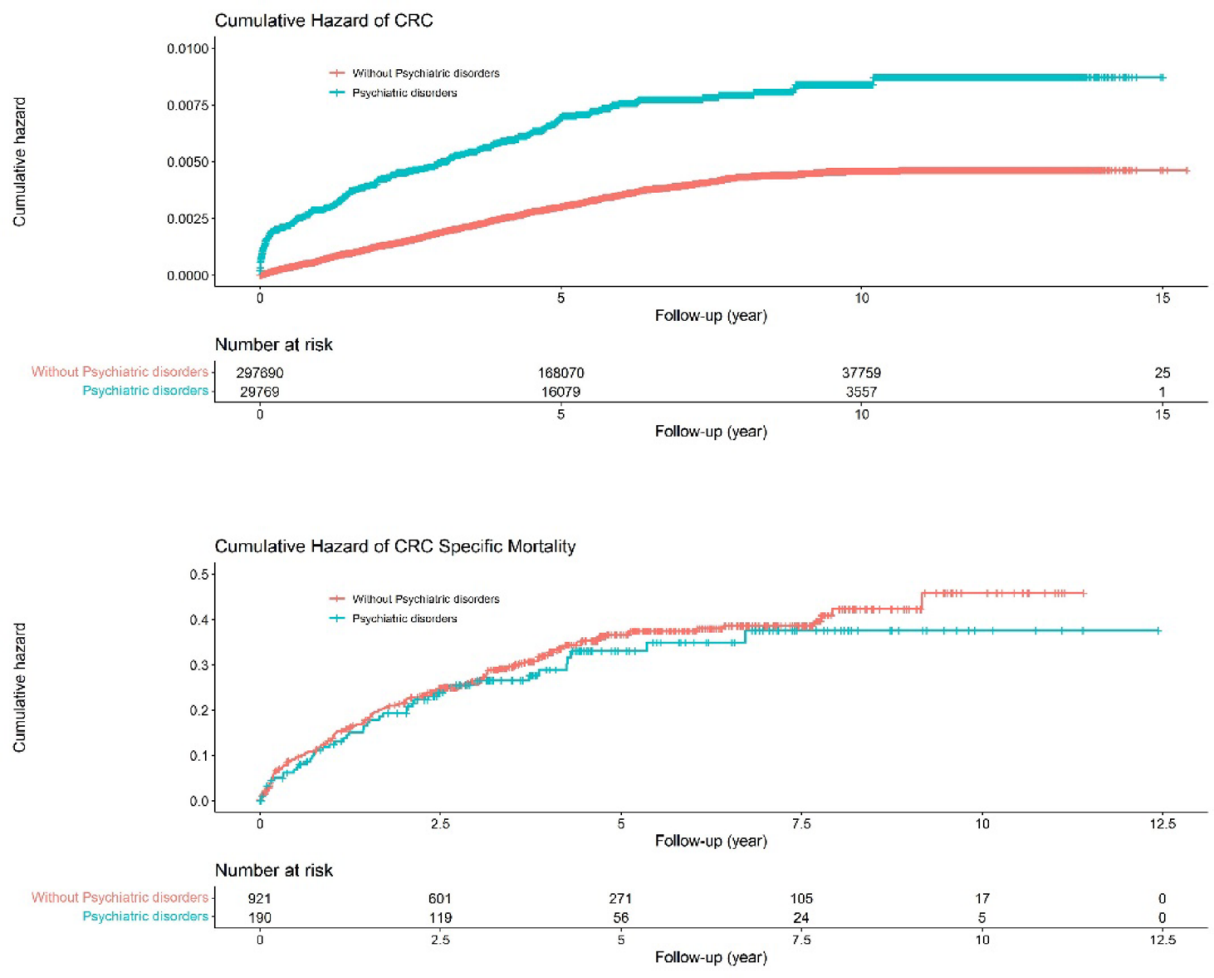
Cumulative hazards of CRC and CRC specific mortality.

After a median follow-up time of 5.69 (IQR: 5.43) years, 190 cases of CRC (115 [60.53%] in men and 75 [39.47%] in women) were recorded among exposed patients (incidence rate, 1.13 per 1000 person-years). Of these cases, 138 (72.63%) were colon tumours and 52 (27.37%) were rectal tumours. There were 921 cases of CRC among the matched unexposed individuals (541 [58.74%] in men and 380 [41.26%] in women; incidence rate, 0.53 per 1000 person-years), Of these cases, 607 (65.91%) were colon tumours and 317 (34.42%) were rectal tumours (Table 2). The 15-year cumulative harzds of CRC was 0.64% (95% CI: 0.55%–0.74%) in the psychiatric patients vs. 0.31% (95% CI: 0.29%–0.33%) in the reference participants (*P* < 0.0001; Fig. 2, upper panel).

The incidence of CRC was clearly higher among patients with psychiatric disorders exposed at an older age (incidence rate, 1.67 per 1000 person-years and 0.58 per 1000 person-years for subjects aged ≥ 60 and < 60 years, respectively), males (incidence rate, 1.51 per 1000 person-years and 0.82 per 1000 person-years for male and female, respectively), and smokers (incidence rate, 1.35 per 1000 person-years and 0.71 per 1000 person-years for ever smokers and never smokers, respectively) (Table 2).

Regarding CRC-specific mortality, 47 deaths from CRC occurred in individuals with psychiatric disorders compared with 246 deaths from CRC in the reference individuals. This represents a CRC death rate of 64.38 per 1000 person-years for patients with psychiatric disorders compared with 71.51 per 1000 person-years in the reference individuals (Supplementary Table 1). The 15-year cumulative hazards of CRC death was 24.74% (95% CI: 18.78%–31.50%) in psychiatric patients vs. 26.71% (95% CI: 23.88%–29.70%) in the reference individuals (*P* = 0.64; Fig. 2, lower panel).

**Table 2:** Absolute incidence rates per 1000 person years (95%CI) of CRC.

	No. of Cancer/No. of Accumulated Person-Years x 1000 Incidence Rare/1000 Person-Years (95%CI)	
	Persons with Psychiatric disorders	Persons without Psychiatric disorders
**ALL**	190/167.81 1.13 (0.98, 1.31)	921/1750.38 0.53 (0.49, 0.56)
**By calendar year at index date**		
2006–2010	28/22.59 1.24 (0.82, 1.79)	148/237.89 0.62 (0.53, 0.73)
2011–2015	116/102.27 1.13 (0.94, 1.36)	616/1065.43 0.58 (0.53, 0.63)
2016–2021	46/42.95 1.07 (0.78, 1.43)	157/447.06 0.35 (0.30, 0.41)
**Age group**		
<60	48/82.86 0.58 (0.43, 0.77)	262/836.11 0.31 (0.28, 0.35)
≧60	142/84.96 1.67 (1.41, 1.97)	659/914.28 0.72 (0.67, 0.78)
**Gender**		
male	115/76.38 1.51 (1.24, 1.81)	541/818.03 0.66 (0.61, 0.72)
female	75/91.44 0.82 (0.65, 1.03)	380/932.35 0.41 (0.37, 0.45)
**Body mass index (BMI)**		
<18.5	0/1.23 0 (0, 2.99)	3/7.84 0.38 (0.08, 1.12)
18.5–24.9	52/47.08 1.1 (0.83, 1.45)	215/589.87 0.36 (0.32, 0.42)
25.0–29.9.0.9	74/66.14 1.12 (0.88, 1.40)	446/742.01 0.6 (0.55, 0.66)
≧30.0	61/51.8 1.18 (0.90, 1.51)	252/403.02 0.63 (0.55, 0.71)
**Smoking Status**		
Ever	149/110.48 1.35 (1.14, 1.58)	456/709.95 0.64 (0.58, 0.70)
Never	40/56.63 0.71 (0.50, 0.96)	462/1034.74 0.45 (0.41, 0.49)
**Drinking Status**		
Ever	181/159.85 1.13 (0.97, 1.31)	887/1672.26 0.53 (0.50, 0.57)
Never	7/7.6 0.92 (0.37, 1.90)	34/76.76 0.44 (0.31, 0.62)
**Meat Consumption**		
Less than twice a week	97/93.96 1.03 (0.84, 1.26)	502/1060.89 0.47 (0.43, 0.52)
2–4 times a week	82/63.66 1.29 (1.02, 1.60)	377/616.52 0.61 (0.55, 0.68)
More than 4 times a week	11/10.08 1.09 (0.54, 1.95)	42/72.2 0.58 (0.42, 0.79)
**Family History**		
Yes	15/15.26 0.98 (0.55, 1.62)	113/151.46 0.75 (0.61, 0.90)
No	175/152.55 1.15 (0.98, 1.33)	808/1598.93 0.51 (0.47, 0.54)
**Aspirin Intake**		
Yes	50/28.33 1.76 (1.31, 2.33)	167/220.32 0.76 (0.65, 0.88)
No	140/139.49 1.00 (0.84, 1.18)	754/1530.07 0.49 (0.46, 0.53)
**Hormone Replacement Therapy**		
Yes	34/36.12 0.94 (0.65, 1.32)	140/282.42 0.5 (0.42, 0.58)
No	41/54.87 0.75 (0.54, 1.01)	239/647 0.37 (0.32, 0.42)
**Diabetes Mellitus**		
Yes	20/11.85 1.69 (1.03, 2.61)	71/73.03 0.97 (0.76, 1.23)
No	170/155.28 1.09 (0.94, 1.27)	848/1673.61 0.51 (0.47, 0.54)

### Psychiatric disorders and CRC risk in the sexes-combined model

**Fig. 3:**
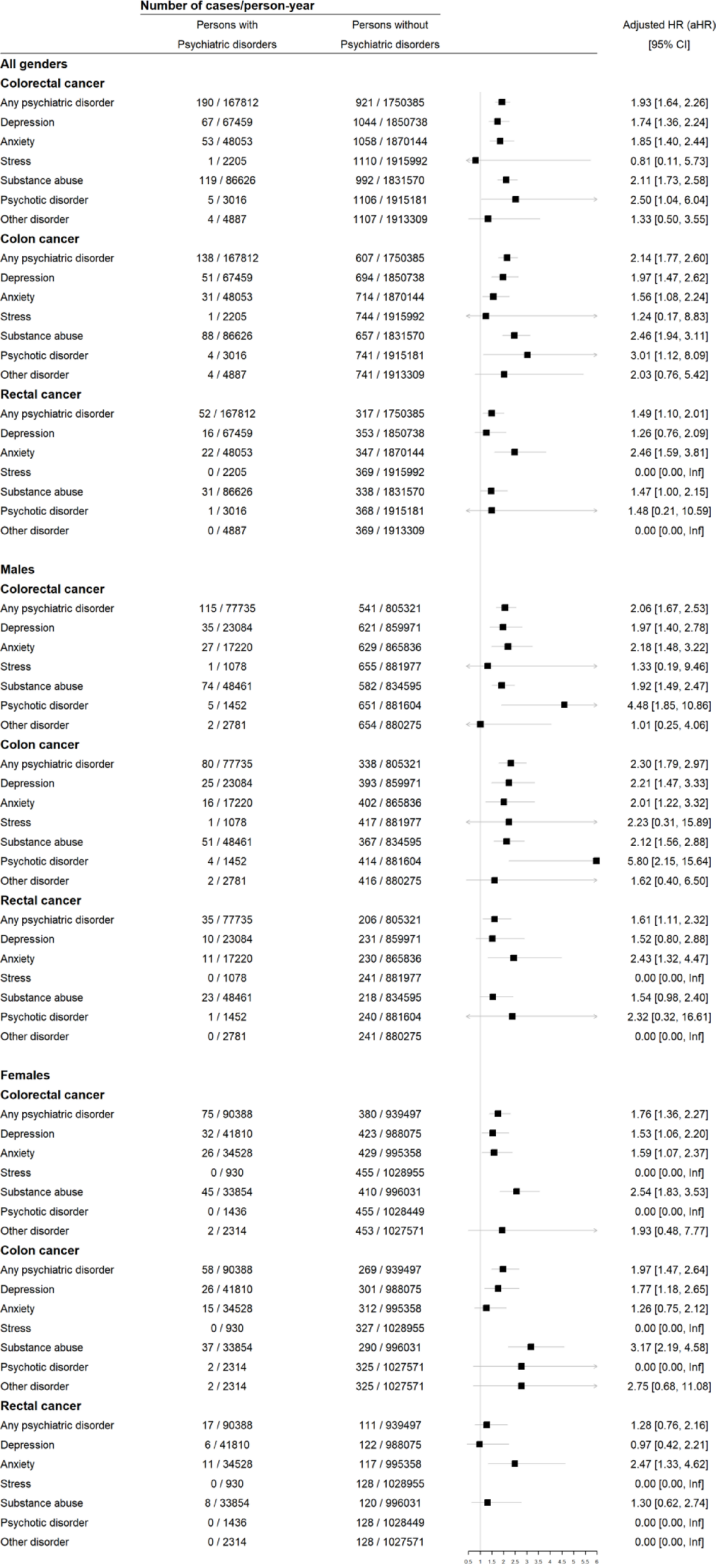
Risk estimates of association between different types of psychiatric disorders and CRC.

In the sexes-combined multivariable model, after controlling for confounders, the risk of CRC was found to be increased among patients with psychiatric disorders compared with matched unexposed individuals in both crude model (crude HR (cHR), 2.14 [95%CI, 1.77–2.43]) and full adjusted model (adjusted HR (aHR), 1.93 [95% CI, 1.64–2.27]) (Supplementary Table 2). Specifically, the aHRs were 1.74 (95% CI, 1.36–2.24) for depression, 1.85 (95% CI, 1.40–2.44) for anxiety, 2.11 (95% CI, 1.73–2.58) for substance abuse, and 2.51 (95% CI, 1.04–6.06) for psychotic disorders. However, after applying the study exclusion criteria, no eligible female cases with stress-related disorders remained in the cohort (0 cases), and only one eligible male case was identified. Consequently, statistical analysis for the association between stress-related disorders and CRC risk was not feasible for this subgroup analysis. (Fig. 3).

For colon cancer, in the sexes-combined model, we observed an elevated hazard in individuals with psychiatric disorders compared with those without such conditions (aHR, 2.15 [95% CI, 1.77–2.60]). For all studied subtypes of psychiatric disorder, the aHRs corroborated the observed associations with CRC. Significant associations were noted for depression (aHR, 1.97 [95% CI, 1.48–2.63]), anxiety (aHR, 1.56 [95% CI, 1.09–2.24]), substance abuse (aHR, 2.46 [95% CI, 1.95–3.12]), and psychotic disorders (aHR, 3.02 [95% CI, 1.12–8.09]) (Fig. 3).

A similarly elevated hazard was observed for rectal cancer among individuals with psychiatric disorders compared with those without such conditions (aHR, 1.49 [95% CI, 1.10–2.01]). Statistically significant associations were found for anxiety (aHR, 2.47 [95% CI, 1.60–3.81]) and substance abuse (aHR, 1.47 [95% CI, 1.00–2.16]) were found, but not for depression, psychotic disorders, or other disorders (Fig. 3).

### Psychiatric disorders and CRC risk among men

In the multivariable models, a pre-existing psychiatric disorder was associated with elevated CRC risk in men(aHR, 2.06 [95% CI, 1.67–2.53]). Among all studied subtypes of psychiatric disorders, a positive relationship was observed for depression (aHR, 1.97 [95% CI, 1.40–2.78]), anxiety (aHR, 2.19 [95% CI, 1.48–3.22]), substance abuse (aHR, 1.92 [95% CI, 1.49–2.47]), and psychotic disorders (aHR, 4.60 [95% CI, 1.90–11.15]).(Fig. 3).

For colon cancer, we observed an elevated hazard among individuals with psychiatric disorders compared with those without such conditions (aHR, 2.31 [95% CI, 1.79–2.97]). The trends for HRs were similar between all the subtypes of psychiatric disorders studied, with the highest HR observed for psychotic disorders (aHR, 5.97 [95% CI, 2.21–16.09]) and significant HRs found for depression (aHR, 2.22 [95% CI, 1.47–3.33]), anxiety (aHR, 2.01 [95% CI, 1.22–3.32]), and substance abuse (aHR, 2.13 [95% CI, 1.57–2.89]). There was no statistical difference for other disorders (Fig. 3).

For rectal cancer, psychiatric disorders were associated with a greater risk compared with those without such conditions (aHR, 1.61 [95% CI, 1.11–2.32]). Although an elevated risk of rectal cancer among individuals with anxiety compared with individuals without anxiety was identified (aHR, 2.43 (95% CI, 1.32–4.47), no such risk was identified for depression, substance abuse, psychotic disorders, or other disorders (Fig. 3).

### Psychiatric disorders and CRC risk among women

The presence of psychiatric comorbidity was associated with an elevated risk of CRC in women (aHR, 1.76 [95% CI, 1.37–2.28]). Significant relationships were observed for all the subtypes of psychiatric disorder studied: depression (aHR, 1.53 [95% CI, 1.07–2.21]), anxiety (aHR, 1.59 [95% CI, 1.07–2.37]), and substance abuse (aHR, 2.55 [(95% CI, 1.83–3.54)). There was no significant difference for psychotic disorders and other disorders (Fig. 3).

For colon cancer, a statistically significant positive association between psychiatric disorder and CRC was identified (aHR, 1.97 [95% CI, 1.47–2.65]). The aHRs were different between all studied subtypes of psychiatric disorders, with significant aHRs for depression (aHR, 1.77 [95% CI, 1.18–2.66]) and substance abuse (aHR, 3.17 [95% CI, 2.19–4.59]). There was no relationship observed for anxiety, psychotic disorders, or other disorders (Fig. 3).

For rectal cancer, no significant difference in risk for individuals with psychiatric disorders was found compared to those without such conditions (aHR, 1.28 [95% CI, 0.76–2.16]). For subgroup analysis, we only observed an elevated risk of rectal cancer among individuals with anxiety compared with individuals without anxiety (aHR, 2.48 (95% CI, 1.33–4.63), but no such association was observed for other types of psychiatric disorders. (Fig. 3).

### Effect modification of the risk of CRC in patients with psychiatric disorders

To test statistical significance of effect modification by sex and cancer location, we computed interaction terms in our main models to analyse whether there were any differences in the association of psychiatric disorders and CRC risk between men and women (aHR, 2.06 [1.67–2.53] vs. 1.76 [1.37–2.28]), as well as colon and rectal cancer (aHR, 2.15 [1.77–2.60] vs. 1.49 [1.10–2.01]). the results indicated that even though the aHRs seem inconsistent, the differences were not statistically significant. (*p* > 0.05 for sex, *p* > 0.1 for cancer location) (Table 3).

**Table 3:** Effect modification of the risk of CRC in patients with psychiatric disorders.

Interaction terms	aHR [95% CI]	*P*-value*
**Sex**		
Male	2.06 [1.67, 2.53]	0.47
Female	1.76 [1.36, 2.27]	
**Cancer location**		
Colon cancer	2.14 [1.77, 2.60]	0.85
Rectal cancer	1.49 [1.10, 2.01]	

*p values were calculated by interaction test by incorportating interaction tern to the main model.

### Association of psychiatric disorders with CRC-specific mortality

Compared to those without psychiatric disorders, no elevated hazard of CRC-specific mortality was observed for those with psychiatric disorders, with an aHR of 0.88 ([95% CI, 0.64–1.22]; *P* = 0.46). Furthermore, no significant risk of CRC-specific mortality was observed for patients with each type of psychiatric disorder compared with individuals without such conditions (Supplementary Fig. 1).

### Association of different number of psychiatric disorders with CRC

For patients with numerous psychiatric disorders, the aHRs for CRC incidence was higher than those with a single disorder (aHR 2.53 [95% CI, 1.9–3.37] vs. aHR 1.78 [95% CI, 1.48–2.13]). Further analysis indicated that this difference between them was statistically significant (*p* < 0.05) (Supplementary-Table 3).

## Discussion

Using the largescale community-based cohort UK Biobank, we found that patients with clinically confirmed psychiatric disorders have an elevated hazard of CRC. Notably, when analysed according to type of psychiatric disorders, patients with depression, anxiety, substance abuse and psychotic disorder, are strongly related to an elevated risk of CRC. Additionally, the risk of CRC in patients with psychiatric disorders is higher for individuals with multiple psychiatric disorders. Finally, there were no significant differences in the association of psychiatric disorders and CRC risk between men and women, as well as colon and rectal cancer. To our knowledge, this was the first largescale study to comprehensive investigate multiple types of psychiatric disorder and their associations with CRC risk in men and women.

Psychiatric disorders such as depression, anxiety, substance abuse, and stress-related disorder are commonly experienced among cancer patients, including CRC patients^15^. Most research into the matter has addressed the influence of mental health problems, especially depression and anxiety, in the period both immediately after cancer diagnosis and later, focusing on survivorship, tumour recurrence, treatment effect, and so on^16,17^. However, other psychiatric disorders have received less attention, and the potential effects of multiple types of psychiatric disorder on subsequent CRC risk has rarely been investigated. Accordingly, in the present study, we focused on patients who were diagnosed with psychiatric disorders before their cancer diagnosis and revealed an elevated hazard of CRC for patients with clinically confirmed psychiatric disorders. These findings highlight the need for CRC monitoring and early prevention and intervention in individuals with mental health problems, thus decreasing their risk of CRC and optimizing the use of healthcare resources and medical expense.

Several causative mechanisms for the association between psychiatric disorder and subsequent CRC risk may be hypothesized. One such mechanism is the activation of the hypothalamic–pituitary–adrenal axis among patients with psychiatric disorders, which has been widely reported^18^. Cortisol, produced by the adrenal cortex, is regulated by the hypothalamus and pituitary gland, and its levels are increased in patients with psychiatric disorders^19^. However, chronically high levels of cortisol have a suppressive effect on T-cell-mediated immune responses^20^, which is associated with an elevated risk of CRC^21^. Furthermore, alterations in cytokine secretion, such as increased levels of the inflammatory markers interleukin-6, TNF-a, and C-reactive protein, which contribute to dysfunctional immune responses, is another possible mechanism by which exposure to psychiatric disorders may be related to CRC risk^22,23^. In addition, poorer healthcare access (except psychiatric care) among patients with psychiatric disorders compared to individuals without might be an additional factor^24^. Similarly, the degree of compliance to medical treatment is lower among patients with psychiatric disorders^25^. Finally, behavioural factors between patients with and without psychiatric disorders are different, and such factors may contribute to the observed association^26^.

Previous research showed that greater exposure to oestrogens in women may predispose them to a lower risk of CRC^24^. We noted a relatively weaker association between psychiatric disorders and subsequent CRC risk in women than in men. However, we also found that receiving HRT will not increase the risk of CRC in patients with or without psychiatric disorders (Table 2). Our further analysis revealed a partial overlap in the 95% CI of the HRs mentioned above. To determine if there was a statistically significant effect modification by sex, we included sex as an interaction term in our main model. The results showed that there was no significant difference of CRC risk between man and women.

CRC is one of the most common cancers worldwide, and approximately one-third of CRCs are rectal cancers and two-thirds of are colon cancers^27^. The embryological origin, anatomy, and function of the colon and rectum are different. Several studies have demonstrated that lifestyle and environmental factors such as physical activity, diet, smoking, and BMI have less impact on rectal cancer than on colon cancer^28,29^. However, our results of interaction terms in main model showed that the cancer location didn’t modify the risk of CRCs on psychiatric disorders.

One explanation for our null finding is that there may actually be no association between psychiatric disorder before CRC diagnosis and its mortality, which seems to be in line with the findings of a previous study, i.e., that the incidence of a psychiatric disorder such as depression before CRC diagnosis does not influence its subsequent mortality^28^. However, psychiatric disorders were confirmed before CRC diagnosis, the remote measurement may dilute its effect on cancer prognosis. Furthermore, the association between psychiatric disorders and CRC-specific mortality is influenced by the disease condition itself, for example, its severity, tumour type, and treatment. The observed dissociation between the elevated incidence of CRC and non-significant trends in mortality requires careful interpretation. Although our findings do not indicate an increase in CRC-specific deaths among individuals with psychiatric disorders, the rising number of diagnoses highlights potential deficiencies in primary prevention or early detection strategies. Implementing targeted monitoring approaches, such as enhancing fecal testing within psychiatric disorders clinics or streamlining colonoscopy referral processes, could provide substantial benefits beyond mortality reduction. Early detection may alleviate the profound impact of late-stage CRC diagnosis on quality of life, including occupational disability, accelerated deterioration of mental health, and increased burdens of physical-psychiatric comorbidities. While overall mortality rates remain stable, earlier intervention can reduce treatment-related morbidity (e.g., less invasive surgeries, fewer emergency presentations) and lower associated healthcare costs. We stress that resource allocation should be tailored to population-specific risks, such as prioritizing high-risk subgroups within psychiatric populations. Integrating CRC screening into existing mental health service frameworks may enhance efficiency without compromising resources allocated to conditions with higher mortality rates. Future cost-effectiveness analyses are warranted to evaluate the feasibility and sustainability of this approach.

The contributions of stress-related psychosocial factors to cancer were studied by Chida et al^30^., who reported that stress and related psychosocial factors are associated with the incidence, survival, and mortality of several types of cancer, including breast cancer, lung cancer, thyroid cancer, lymphoid or hematopoietic cancer, hepatobiliary cancer, and head and neck cancer, but not with CRC. Additionally, a large Swedish cohort study by Song et al. found no statistically significant association between stress-related disorders and CRC risk^31^. Other studies have similarly reported non-significant associations^32–34^.Our research also explored the association between stress-related disorders and CRC risk and mortality; however, the null finding obtained in this study regarding their relationship may be a consequence of insufficient case numbers (only 1.19% of all stress-related disorder/psychiatric disorders). Further cohort studies with higher numbers of stress-related-disorder patients are required to provide stronger scientific evidence.

Notably, the highest risk estimates were observed for psychotic disorders among the psychiatric subtypes examined. This could be attributed to several factors potentially more prevalent or severe in this population, including a greater burden of chronic inflammation^21,35^, significant metabolic adverse effects from long-term antipsychotic medication use^36,37^, and profound disparities in accessing preventive healthcare^38^. However, this finding must be interpreted with caution due to the small number of individuals with psychotic disorders and the consequent limited number of CRC events in this subgroup, which leads to wide confidence intervals and potential overestimation of the effect size. Future studies with larger cohorts of individuals with severe mental illness are required to obtain a more precise estimate of the CRC risk associated with psychotic disorders and to disentangle the contributions of the disease itself, its treatment, and associated healthcare disparities.

A major strength of our study is its largescale, population-based cohort design and the complete follow-up. Another important strength is the fact that the data on psychiatric disorder and CRC were collected prospectively and independently, which minimizes information bias. Furthermore, the data in UK Biobank enabled comprehensive case ascertainment and allowed a broad range of potential confounding variables to be analysed for all subgroups.

A potential shortcoming of our study is that UK Biobank has some inherent limitations, with most of the participants being white (94.6%)^39^. Additionally, the UKB recruited participants between 2006 and 2010, with the majority of participants aged 40–69 at the time of recruitment, resulting in an overall older age distribution. Therefore, the study may not accurately represent the general population, particularly with regard to under-representation of early-onset psychiatric morbidity (peaking in the 20 s). Moreover, because most of the variables for psychiatric patients who received their first diagnosis before June 2006 were unavailable, the focus of this study is inevitably on new-onset psychiatric patients who received their first diagnosis of a disorder between June 1, 2006, and December 15, 2021, thus raising concerns about potential selection bias. Of note, this research is based on cohort studies using UK biobank database, by formulating standardized data extraction and collection rules, the timing of diagnosis of psychiatric disorders and CRC could be clarified. However, the UKB is based on real-world data collection, whether a patient seeks medical care or even receives treatment is influenced by many factors (e.g., financial status, severity of disease, perception, etc.). Cancer cases were identified through linkage to national cancer registries, which might be subject to misclassification or underreporting. Thus, it is true that we cannot exclude potentially undiagnosed CRC patients prior to those diagnosed with psychiatric disorder, nor can we rule out the balance of this distribution among participants with or without psychiatric disorder. Based on a single-center database, we were unable to address this type of selection bias. The proof of causality is not as strong as that of high-quality prospective RCT studies. In the future, higher-quality prospective RCT research is required to provide stronger scientific evidence. But this does not obscure the merits of database-based research that helps us to explore the long-term effects of exposure, which we believe is very helpful in this field. Finally,

our study focused on patients with clinically confirmed psychiatric disorders diagnosed at the inpatient hospita. While we acknowledge that restricting exposure to hospital-based diagnoses excludes some patients diagnosed in primary care settings and may result in control group misclassification, we believe this approach is critical for concentrating on severe and persistent psychiatric disorders. This strategy enhances diagnostic accuracy and reduces heterogeneity and potential biases that could arise from including numerous transient or mild conditions. Many primary care cases involve temporary psychological disturbances or subclinical conditions triggered by acute stressors such as work pressure or adverse life events. These conditions are generally less severe, shorter in duration, and may resolve through self-regulation, social support, brief counseling, or lifestyle modifications. Theoretically, the resulting control group misclassification is more likely to lead to an underestimation of the association strength rather than introducing spurious associations. This limitation should be carefully considered when interpreting our findings.

In conclusion, this largescale, population-based cohort study, conducted using UK Biobank participants, indicated that patients with psychiatric disorders are at elevated hazard of CRC. Although the underlying causative mechanism for this association requires further investigation, the mortality rate of CRC among individuals with psychiatric disorders has not increased. However, the rising incidence of CRC diagnoses indicates a potential need for targeted health services. This could involve integrating this population into existing CRC screening programs, such as fecal immunochemical testing and colonoscopy surveillance, to facilitate earlier detection and intervention.

## Supplementary Information

Below is the link to the electronic supplementary material.

Supplementary Material 1

## Data Availability

We thank the UK Biobank participants and the UK Biobank team for their dedication in collecting and curating this invaluable dataset, and for making it available to the research community. The datasets generated and/or analysed during the current study are available in the UK Biobank repository. [https://www.ukbiobank.ac.uk/](https:/www.ukbiobank.ac.uk)This research was done using the UK Biobank Resource under Application 77512.
