# Supplementary material for: Psychiatric disorders associated with increased risk of colorectal cancer in the UK biobank cohort
Source: Sci Rep. 2026 Jan 13;16:1597. doi: 10.1038/s41598-025-31083-1 (PMC12800037; doi:10.1038/s41598-025-31083-1)

**Supplementary Files**

**Psychiatric Disorders Associated with Increased Risk of Colorectal Cancer in the UK Biobank Cohort**

**Zijing Wang^1#^, Tingxi Zhu^2，5#^, Litao Huang^4^, Xian Zhang^1^, Xiuhe Lv^1^, Xiaoshuang Zhang^1^, Li Yang^1^, David Kerr^3^, Jinlin Yang^1*^**

**This file contains Supplementary table 1-3 and Supplementary Figure 1-2**

**Supplementary table 1. Absolute incidence rates per 1000 person years (95%CI) of CRC specific mortality in patients with psychiatric disorders and reference individuals.**

|  | **No. of Death/No. of Accumulated Person-Years x 1000 Mortality Rate/1000 Person-Years** | |
| --- | --- | --- |
|  | Persons with Psychiatric disorders | Persons without Psychiatric disorders |
| **ALL** | 47 / 0.73  64.38 ( 47.69 , 84.7 ) | 246 / 3.44  71.51 ( 63.12 , 80.64 ) |
| **By calendar year at diagnosed date** |  |  |
| 2006-2010 | 0 / 0.04  0 ( 0 , 88.1 ) | 2 / 0.07  28.57 ( 3.48 , 99.43 ) |
| 2011-2015 | 31 / 0.39  79.49 ( 54.64 , 110.93 ) | 92 / 1.71  53.8 ( 43.59 , 65.58 ) |
| 2016-2021 | 16 / 0.3  53.33 ( 30.79 , 85.17 ) | 152 / 1.66  91.57 ( 78.12 , 106.47 ) |
| **Age group** |  |  |
| <60 | 8 / 0.22  36.36 ( 15.83 , 70.39 ) | 70 / 1.05  66.67 ( 52.34 , 83.48 ) |
| ≧60 | 39 / 0.52  75 ( 53.87 , 101.1 ) | 176 / 2.39  73.64 ( 63.49 , 84.85 ) |
| **Gender** |  |  |
| male | 25 / 0.45  55.56 ( 36.27 , 80.92 ) | 149 / 1.97  75.63 ( 64.34 , 88.21 ) |
| female | 22 / 0.29  75.86 ( 48.15 , 112.61 ) | 97 / 1.47  65.99 ( 53.83 , 79.91 ) |
| **Body mass index (BMI)** |  |  |
| <18.5 | 0 / 0  NaN | 0 / 0.01  0 ( 0 , 308.5 ) |
| 18.5-24.9 | 12 / 0.2  60 ( 31.38 , 102.46 ) | 52 / 0.83  62.65 ( 47.14 , 81.35 ) |
| 25.0-29.9 | 18 / 0.31  58.06 ( 34.77 , 90.22 ) | 117 / 1.67  70.06 ( 58.28 , 83.37 ) |
| ≧30.0 | 15 / 0.21  71.43 ( 40.53 , 115.07 ) | 77 / 0.9  85.56 ( 68.11 , 105.77 ) |
| **Smoking Status** |  |  |
| Ever | 38 / 0.58  65.52 ( 46.78 , 88.82 ) | 113 / 1.69  66.86 ( 55.42 , 79.84 ) |
| Never | 8 / 0.15  53.33 ( 23.3 , 102.38 ) | 133 / 1.73  76.88 ( 64.76 , 90.45 ) |
| **Drinking Status** |  |  |
| Ever | 45 / 0.71  63.38 ( 46.6 , 83.89 ) | 235 / 3.32  70.78 ( 62.29 , 80.04 ) |
| Never | 2 / 0.02  100 ( 12.35 , 316.98 ) | 11 / 0.12  91.67 ( 46.65 , 158.1 ) |
| **Meat Consumption** |  |  |
| Less than twice a week | 23 / 0.39  58.97 ( 37.75 , 87.18 ) | 125 / 1.82  68.68 ( 57.49 , 81.29 ) |
| 2-4 times a week | 23 / 0.29  79.31 ( 50.94 , 116.63 ) | 105 / 1.51  69.54 ( 57.22 , 83.55 ) |
| More than 4 times a week | 1 / 0.05  20 ( 0.51 , 106.47 ) | 16 / 0.11  145.45 ( 85.49 , 225.44 ) |
| **Family History** |  |  |
| Yes | 2 / 0.08  25 ( 3.04 , 87.41 ) | 27 / 0.44  61.36 ( 40.82 , 88.03 ) |
| No | 45 / 0.66  68.18 ( 50.16 , 90.17 ) | 219 / 3  73 ( 63.94 , 82.9 ) |
| **Aspirin Intake** |  |  |
| Yes | 11 / 0.19  57.89 ( 29.25 , 101.23 ) | 53 / 0.57  92.98 ( 70.43 , 119.86 ) |
| No | 36 / 0.54  66.67 ( 47.13 , 91.1 ) | 193 / 2.87  67.25 ( 58.36 , 77.03 ) |
| **Hormone Replacement Therapy** |  |  |
| Yes | 8 / 0.14  57.14 ( 24.99 , 109.49 ) | 39 / 0.53  73.58 ( 52.85 , 99.22 ) |
| No | 14 / 0.15  93.33 ( 51.97 , 151.64 ) | 58 / 0.93  62.37 ( 47.69 , 79.88 ) |
| **Diabetes Mellitus** |  |  |
| Yes | 7 / 0.06  116.67 ( 48.21 , 225.72 ) | 18 / 0.22  81.82 ( 49.21 , 126.23 ) |
| No | 40 / 0.67  59.7 ( 42.99 , 80.41 ) | 227 / 3.22  70.5 ( 61.89 , 79.89 ) |

**Supplementary Table 2. HRs for CRC incidence during all progressively models in patients with psychiatric disorders and matched cohort.**

| **HRs for CRC incidence during all progressively models in patients with psychiatric disorders and matched cohort** | | | | |
| --- | --- | --- | --- | --- |
|  | HRs (95% CI) | | | |
|  | Model1 Crude model | Model2 Adding sociodemographic covariates | Model3 Adding health status covariates | Model4 Full Model Adding behavioral covariates |
| **Psychiatric disorders** | 2.14 [1.83, 2.51] | 2.07 [1.77, 2.43] | 2.03 [1.73, 2.39] | 1.93 [1.64, 2.26] |
| **Age group, No. (%)** |  |  |  |  |
| <60 | - | Ref | Ref | Ref |
| ≧60 |  | 2.03 [1.76, 2.33] | 1.96 [1.69, 2.26] | 1.94 [1.68, 2.24] |
| **Gender, No. (%)** |  |  |  |  |
| male | - | Ref | Ref | Ref |
| female |  | 0.59 [0.52, 0.66] | 0.42 [0.06, 3.02] | 0.45 [0.06, 3.23] |
| **Race or Ethnicity, No. (%)** |  |  |  |  |
| White | - | Ref | Ref | Ref |
| Black |  | 1.19 [0.76, 1.87] | 1.14 [0.72, 1.79] | 1.20 [0.76, 1.89] |
| Asian |  | 0.34 [0.18, 0.64] | 0.33 [0.18, 0.62] | 0.36 [0.19, 0.68] |
| Others |  | 0.88 [0.52, 1.50] | 0.87 [0.51, 1.47] | 0.87 [0.51, 1.49] |
| Unknown |  | 2.49 [1.37, 4.53] | 2.50 [1.38, 4.54] | 2.51 [1.38, 4.57] |
| **townsend, median (IQR)** | - | 0.99 [0.97, 1.01] | 0.99 [0.97, 1.01] | 0.99 [0.97, 1.01] |
| **Educational Attainment, No. (%)** |  |  |  |  |
| College or University degree | - | Ref | Ref | Ref |
| A levels/AS levels or equivalent | - | 1.12 [0.90, 1.38] | 1.10 [0.89, 1.36] | 1.08 [0.88, 1.34] |
| O levels/GCSEs or equivalent | - | 1.13 [0.95, 1.35] | 1.10 [0.93, 1.31] | 1.08 [0.90, 1.28] |
| CSEs or equivalent | - | 0.98 [0.73, 1.31] | 0.95 [0.71, 1.27] | 0.93 [0.70, 1.25] |
| NVQ or HND or HNC or equivalent | - | 0.93 [0.72, 1.21] | 0.90 [0.69, 1.17] | 0.87 [0.67, 1.13] |
| Other professional qualifications | - | 1.26 [0.96, 1.65] | 1.22 [0.93, 1.60] | 1.20 [0.92, 1.58] |
| None of the above | - | 1.11 [0.92, 1.35] | 1.06 [0.87, 1.28] | 1.02 [0.84, 1.24] |
| Prefer not to answer | - | 1.68 [1.15, 2.45] | 1.64 [1.12, 2.39] | 1.60 [1.10, 2.33] |
| **Household Annual Income, No. (%)** |  |  |  |  |
| Less than 18,000 | - | Ref | Ref | Ref |
| 18,000 to 30,999 | - | 0.85 [0.71, 1.02] | 0.86 [0.72, 1.03] | 0.86 [0.72, 1.03] |
| 31,000 to 51,999 | - | 0.79 [0.65, 0.95] | 0.80 [0.66, 0.97] | 0.80 [0.66, 0.97] |
| 52,000 to 100,000 | - | 0.76 [0.61, 0.94] | 0.77 [0.62, 0.96] | 0.77 [0.62, 0.96] |
| Greater than 100,000 | - | 0.68 [0.47, 0.98] | 0.70 [0.48, 1.01] | 0.71 [0.49, 1.02] |
| Do not know | - | 1.03 [0.76, 1.40] | 1.04 [0.77, 1.40] | 1.05 [0.77, 1.42] |
| Prefer not to answer | - | 0.89 [0.71, 1.11] | 0.90 [0.72, 1.13] | 0.91 [0.73, 1.14] |
| **Body mass index (BMI), No. (%)** |  |  |  |  |
| <18.5 | - | - | Ref | Ref |
| 18.5-24.9 | - | - | 1.26 [0.40, 3.93] | 1.26 [0.40, 3.95] |
| 25.0-29.9 | - | - | 1.66 [0.53, 5.16] | 1.65 [0.53, 5.14] |
| ≧30.0 | - | - | 1.66 [0.53, 5.19] | 1.64 [0.53, 5.12] |
| Unknown | - | - | 2.05 [0.54, 7.73] | 2.04 [0.54, 7.72] |
| **Family History, No. (%)** |  |  |  |  |
| Yes | - | - | Ref | Ref |
| No | - | - | 0.80 [0.66, 0.96] | 0.80 [0.66, 0.96] |
| **Aspirin Intake, No. (%)** |  |  |  |  |
| Yes | - | - | Ref | Ref |
| No | - | - | 0.87 [0.74, 1.02] | 0.88 [0.75, 1.03] |
| **Hormone Replacement Therapy, No. (%)** |  |  |  |  |
| Yes | - | - | Ref | Ref |
| No | - | - | 1.05 [0.87, 1.28] | 1.06 [0.88, 1.29] |
| Unknown | - | - | 0.70 [0.10, 5.00] | 0.71 [0.10, 5.10] |
| **Diabetes Mellitus, No. (%)** |  |  |  |  |
| Yes | - | - | Ref | Ref |
| No | - | - | 0.73 [0.58, 0.91] | 0.73 [0.58, 0.92] |
| Unknown | - | - | 0.52 [0.13, 2.10] | 0.52 [0.13, 2.12] |
| **Smoking Status, No. (%)** |  |  |  |  |
| Ever | - | - | - | Ref |
| Never | - | - | - | 0.77 [0.68, 0.88] |
| Unknown | - | - | - | 0.80 [0.30, 2.15] |
| **Drinking Status, No. (%)** |  |  |  |  |
| Ever | - | - | - | Ref |
| Never | - | - | - | 1.01 [0.73, 1.39] |
| Unknown | - | - | - | 1.79 [0.44, 7.25] |
| **Meat Consumption, No. (%)** |  |  |  |  |
| Less than twice a week | - | - | - | Ref |
| 2-4 times a week | - | - | - | 1.15 [1.02, 1.30] |
| More than 4 times a week | - | - | - | 1.03 [0.77, 1.37] |
| Unknown | - | - | - | 0.00 [0.00, Inf] |

**Supplementary Table 3: Risk of CRC among individuals with different number of psychiatric disorders.**

|  | **No. of Cancer/No. of Accumulated Person-Years x 1000 Incidence Rare/1000 Person-Years (95%CI)** | | | |
| --- | --- | --- | --- | --- |
|  | **Persons with Psychiatric disorders** | **Persons without Psychiatric disorders** | **Hazard Ratio (95% CI)** | **p value** |
|  |  |  |  |  |
| **Number of diagnosed psychiatric disorders** | | |  |  |
| 1 | 139/131.03 1.06 (0.89 , 1.25) | 921/1750.38 0.53 (0.49, 0.56) | 1.77  (1.48, 2.13) | 0.032 |
| ≧2 | 51/36.78 1.39 (1.03 , 1.82) | 921/1750.38 0.53 (0.49, 0.56) | 2.53  (1.90, 3.36) |  |

**Supplementary figure 1. Risk estimates of association between different types of psychiatric disorders and CRC specific mortality, in patients with psychiatric disorders and matched general population comparators.**


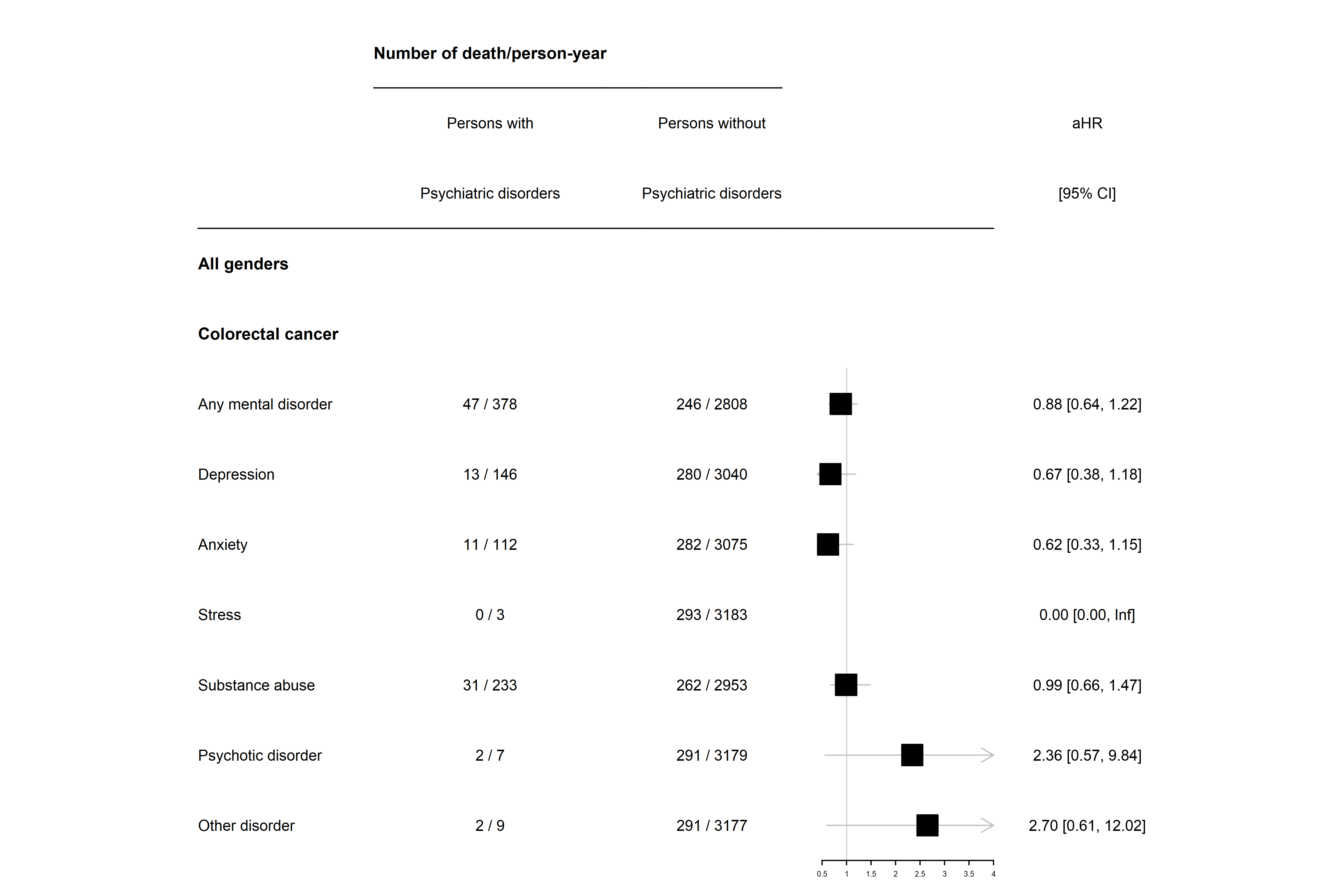


**Supplementary figure 2. Cumulative Incidence Function of CRC in Patients with or without Psychiatric Disorders: A Comparison with the Kaplan-Meier Estimate**


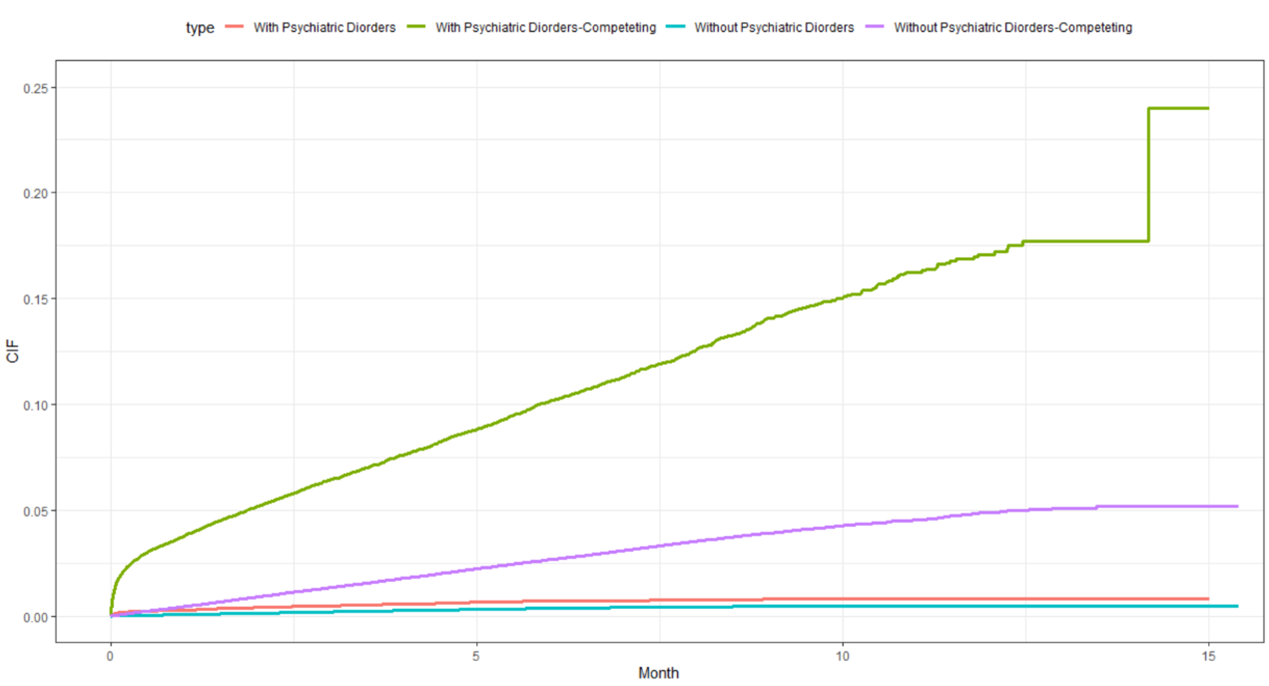

Supplement: Supplementary file 1 — Supplementary Material 1 [file 41598_2025_31083_MOESM1_ESM.docx]
